# Nicorandil versus nitroglycerin: a pilot study

**DOI:** 10.1186/cc9508

**Published:** 2011-03-11

**Authors:** V Singh, S Momin, B Shah

**Affiliations:** 1 Addenbrooke's NHS Foundation Trust, Cambridge, UK; 2 West Suffolk Hospital NHS Trust, Bury St Edmunds, UK; 3 NHL Medical college, Ahmedabad, India

## Introduction

Continuous exposure to nitrates is associated with tachyphylaxis. This study compares the effects and tolerance during intravenous treatment with nitroglycerin and nicorandil over a 48-hour period.

## Methods

Twenty patients with congestive heart failure and pulmonary capillary wedge pressure (PCWP) ≥18 mmHg were randomly assigned to nitroglycerin or nicorandil intravenous infusions. Doses were titrated to obtain a reduction of PCWP of at least 30% at 6 hours and then maintained for 48 hours.

## Results

There was no statistical difference between the groups in terms of age, sex, and NYHA grade. The pretreatment PCWP for nitroglycerin was 25.7 mmHg, decreasing to 18.4 mmHg at 6 hours. The values for nicorandil were 25.4 mmHg and 17.3 mmHg, respectively. There was no statistical difference between the two groups (*P *= 0.79 pretreatment and 0.23 at 6 hours). The mean PCWP values for 24 hours were 19.7 and 17.4, respectively, which was statistically significant (*P *= 0.036). Similarly, the values for 48 hours were 20.6 and 17.9, which was significant (*P *= 0.026) (see Table 1).

**Table 1:** PCWP values before and after treatment

Variable	Nitroglycerin	Nicorandil
Number	10 (8/2)	10 (7/3)
Age	49.9	51.4
Pretreatment	25.7	25.4
6 hours	18.4	17.3
24 hours	19.7	17.4
48 hours	20.6	17.9

## Conclusions

Intravenous nicorandil administration gives similar reductions in PCWP compared with nitroglycerin with significantly less haemodynamic tolerance over a 48-hour period compared with nitroglycerin. This might represent a clinical advantage of nicorandil in the short-term treatment of patients with congestive heart failure.
